# LncRNA MEG8 promotes TNF-α expression by sponging miR-454-3p in bone-invasive pituitary adenomas

**DOI:** 10.18632/aging.203048

**Published:** 2021-05-19

**Authors:** Hai-Bo Zhu, Bin Li, Jing Guo, Ya-Zhou Miao, Yu-Tao Shen, Ya-Zhuo Zhang, Peng Zhao, Chu-Zhong Li

**Affiliations:** 1 Beijing Neurosurgical Institute, Capital Medical University, Fengtai 100070, Beijing, China; 2 Department of Neurosurgery, Beijing Tiantan Hospital, Capital Medical University, Fengtai 100070, Beijing, China; 3 Beijing Institute for Brain Disorders Brain Tumor Center, Fengtai 100070, Beijing, China; 4 China National Clinical Research Center for Neurological Diseases, Fengtai 100070, Beijing, China

**Keywords:** BIPAs, ceRNAs, TNF-α, lncRNA MEG8, miR-454-3p

## Abstract

There are few studies on the mechanism of pituitary adenoma (PA) destroying bone. The current study aimed to investigate the role of MEG8/miR-454-3p/TNF-α in bone-invasive pituitary adenomas (BIPAs). In this study, we report that lncRNA MEG8 and TNF-α are upregulated in BIPA tissues while miR-454-3p is downregulated, which is associated with poor progression-free survival (PFS). Functional assays revealed the role of up-regulated MEG8 and down-regulated miR-454-3p in promoting bone destruction. Mechanistically, MEG8 promotes TNF-α expression by sponging miR-454-3p, which ultimately leads to the occurrence of bone destruction. The mechanism is confirmed *in vivo* and *in vitro*. Therefore, our data illustrated a new regulatory mechanism of MEG8/miR-454-3p/TNF-α in BIPAs. It may provide a useful strategy for diagnosis and treatment for BIPA patients.

## INTRODUCTION

Pituitary adenoma is one of the most common tumors in the central nervous system. Although pituitary adenoma is a benign tumor, invasive pituitary adenoma (IPA) significantly affected the prognosis of patients. Bone-invasive pituitary adenoma (BIPA) is a type of invasive pituitary adenoma. BIPA accounted for 6.4% of all types of pituitary adenoma in our previous research [1]. BIPAs damage important bone anatomy, and the difficulty and risk of surgery, tumor recurrence rate and complication rate are significantly increased. But there are very few studies on BIPAs.

The destruction of bone is inseparable from the differentiation and maturation of osteoclasts. Tumor necrosis factor-alpha (TNF-α) can acts on osteoclast precursor through paracrine and activates MAPK / NFATc1 pathway to promote osteoclast differentiation and maturation, which is identified in previous studies [1]. TNF-α is a dominant cytokine that plays a critical role in the promotion of pathologic osteoclast formation leading to inflammatory bone destruction [2]. TNF-α can induce osteoclast differentiation by activating MAPK and NF-kB pathways [3] and promote the expression of osteolytic cytokines, such as M-CSF and RNAKL [4]. The regulatory mechanism of TNF-α in BIPAs is currently unclear.

It has been revealed that competing endogenous RNAs (ceRNAs) play an important role in the progression and occurrence of tumors [5]. Long noncoding RNAs (lncRNAs) promote protein-coding mRNAs expression by sponging microRNA (miRNA), which is one of the most classic gene regulation networks [6]. It has been confirmed that lncRNA MEG8 and miR-454-3p associated with TNF-α expression in BIPAs in previous studies [1]. Recent studies found MEG8 and miR-454-3p were abnormal in many cancers. Aberrant expression of MEG8 has been found associated with progression of tumors. It has been reported that MEG8 contributes to epigenetic progression of the epithelial-mesenchymal transition of lung and pancreatic cancer cells [7]. It has been found that miR-454-3p is downregulated in cervical cancer [8], bladder cancer [9], renal carcinoma [10], chondrosarcoma [11] and glioblastoma [12].

In the present study, we explored the role of MEG8/miR-454-3p/TNF-α in BIPAs. It may provide a useful strategy for diagnosis and treatment for BIPA patients.

## RESULTS

### Highly expressed TNF-α in BIPAs is associated with poor prognosis

**Figure 1:**
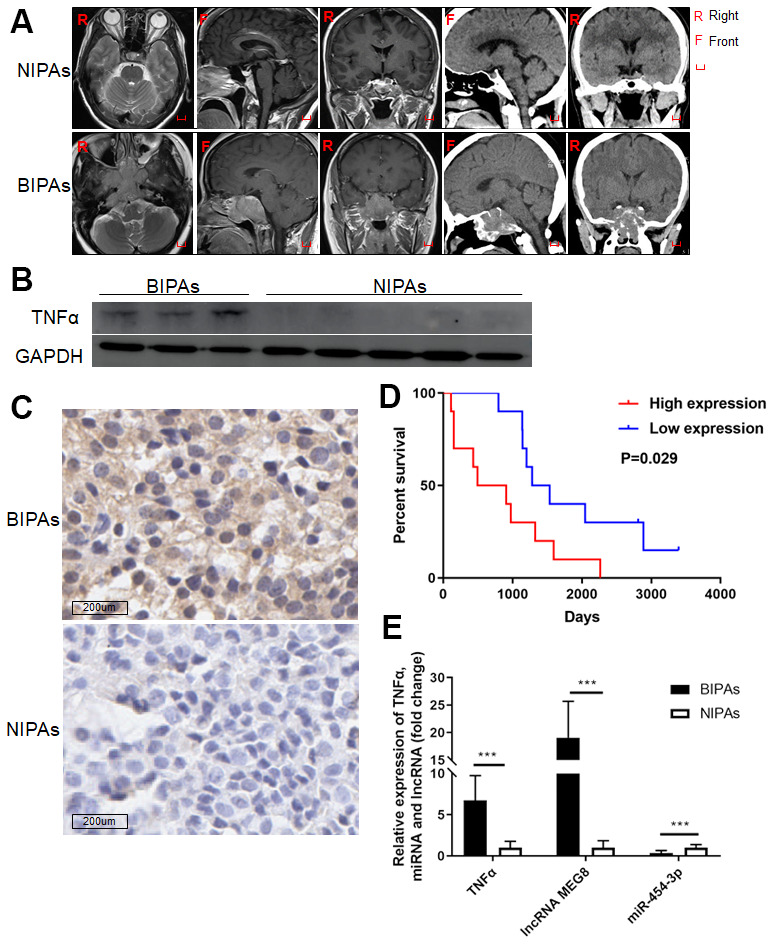
**The expression of TNF-α, MEG8 and miR-454-3p in PAs and the relationship between the expression of TNF-α and the prognosis of patients.** (**A**) The preoperative images of NIPAs and BIPAs. Western blot (**B**) and IHC (**C**) were used to detect the expression of TNF-α in BIPAs and NIPAs. (**D**) Kaplan-Meier plotter was used to analyze the regrowth-free curves of patients in TNF-α high/low groups. (**E**) RT-qPCR was used to detect the expression of TNF-α, MEG8 and miR-454-3p in BIPAs and NIPAs. ***P < 0.001.

The determination of BIPAs depends on preoperative images (Figure 1A), bone destruction observed during operation or pathological reports showing bone tissue discontinuity. Western blot and IHC show TNF-α are upregulated in BIPA tissues compared with NIPAs (Figure 1B, 1C). Highly expressed TNF-α in BIPAs was associated with poor prognosis (Figure 1D).

### MEG8 and TNF-α are upregulated in BIPA tissues while miR-454-3p is downregulated

The expression of MEG8, miR-454-3p and TNF-α were examined by RT-qPCR in twenty BIPAs and twenty NIPAs, finding that MEG8 and TNF-α are upregulated in BIPA tissues while miR-454-3p is downregulated (p<0.001, Figure 1E).

### The expressions of MEG8 and miR-454-3p, miR-454-3p and TNF-α are negatively correlated, MEG8 and TNF-α are positively correlated

**Figure 2:**
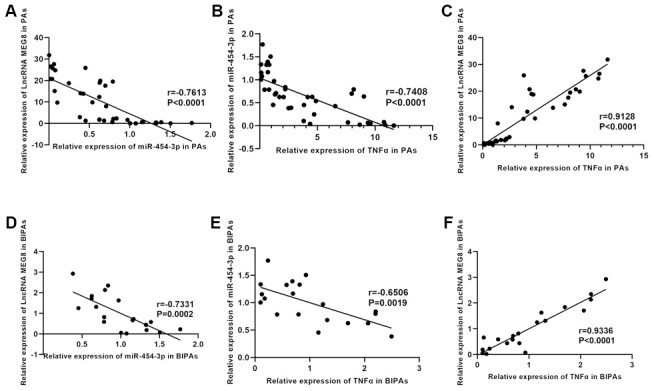
**The relationship between TNF-α expression and prognosis and the correlation between TNF-α, MEG8 and miR-454-3p.** The correlation between MEG8 and miR-454-3p (**A**), miR-454-3p and TNF-α (**B**), MEG8 and TNF-α (**C**) in PAs was analyzed according to RT-qPCR results. The correlation between MEG8 and miR-454-3p (**D**), miR-454-3p and TNF-α (**E**), MEG8 and TNF-α (**F**) in BIPAs was analyzed according to RT-qPCR results.

The correlation between MEG8 and miR-454-3p expression, miR-454-3p and TNF-α expression, MEG8 and TNF-α in pituitary adenomas were analyzed according to RT-qPCR results. There is a negative correlation between the MEG8 and miR-454-3p (p<0.0001, r=-0.7613) (Figure 2A) at RNA expression levels in PAs. In PAs, there is a negative correlation between the miR-454-3p and TNF-α (p<0.0001, r=-0.7408) (Figure 2B). A positive correlation was observed between the MEG8 and TNF-α (p<0.0001, r=0.9128) (Figure 2C). In BIPAs, there is a negative correlation between the MEG8 and miR-454-3p (p=0.0001=2, r=-0.7331) (Figure 2D), a negative correlation was observed between the miR-454-3p and TNF-α (p=0.0019, r=-0.6506) (Figure 2E) and a positive correlation was observed between the MEG8 and TNF-α (p<0.0001, r=0.9336)(Figure 2F).

### TNF-α is a target gene of miR-454-3p

**Figure 3:**
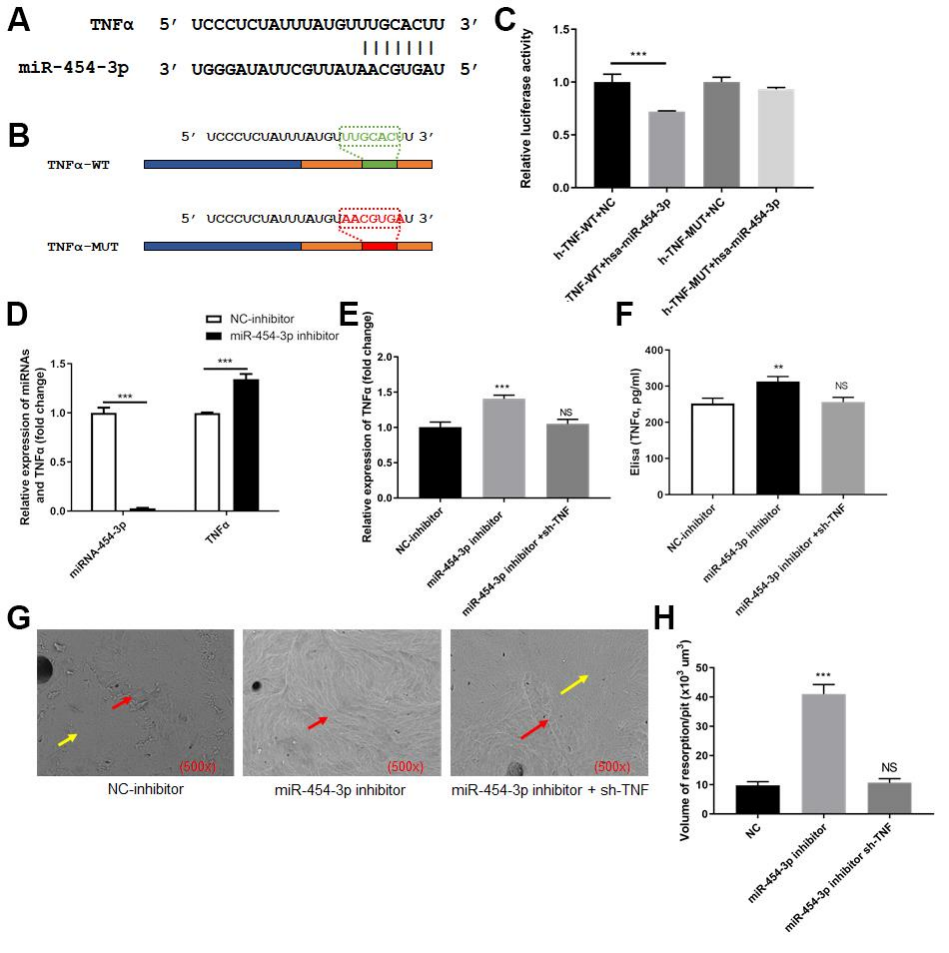
**TNF-α was targeted by miR-454-3p in 293T cells.** (**A**) The predicted binding site between TNF-α and miR-454-3p. (**B**) The predicted miR-454-3p binding site (TNF-α-wt) and its matched mutant site (TNF-α-mut). (**C**) Luciferase activity detection. (**D**) RT-qPCR analyses measured relative miR-454-3p and TNF-α expression levels. (**E**) RT-qPCR analyses measured TNF-α expression levels. (**F**) Elisa analyses measured TNF-α expression levels. (**G**) Scanning electron microscope results of bone slices (500x). (**H**) Volume of resorption/pit (x10^3^ um^3^). **P < 0.01, ***P < 0.001.

We focused on TNF-α and attempted to clarify whether TNF-α was a target of miR-454-3p. We predicted the binding site between TNF-α and miR-454-3p based on data from the StarBase and TargetScan. The predicted binding site between TNF-α and miR-454-3p was presented in Figure 3A. We constructed luciferase reporter plasmids containing the predicted miR-454-3p binding site (TNF-α-wt) and its matched mutant site (TNF-α-mut) (Figure 3B). Co-transfection of miR-454-3p and TNF-α-wt dramatically reduced the luciferase activities, while co-transfection with miR-454-3p and TNF-α-mut had no influence on the luciferase activities (p<0.001, Figure 3C).

### Inhibiting miR-454-3p increases the expression of TNF-α and further promotes RAW264.7 cells to destroy bone

After miR-454-3p was knocked down by its inhibitor in 293T cells, the expression of TNF-α increased significantly (p<0.001, Figure 3D). Enzyme-linked immunosorbent assay (ELISA) also indicated that the amount of TNF-α in the medium increased after miR-454-3p was knocked down (p<0.01, Figure 3F). We use cell and bone slices co-culture experiments to study the changes in cell's osteoclast ability. When miR-454-3p of 293T cells in the lower chamber of transwell was knocked down, the expression of TNF-α increased, which resulted in the bone-breaking ability of RAW264.7 cells was enhanced in the upper chamber of transwell (Figure 3G), and the bone-breaking area of RAW264.7 cells was increased significantly (p<0.001, Figure 3H). Moreover, we added additional groups to conduct rescue experiments (Figure 3E–3H). These results further confirmed that inhibiting miR-454-3p increases the expression of TNF-α and further promotes RAW264.7 cells to destroy bone.

### MiR-454-3p is a target gene of MEG8

**Figure 4:**
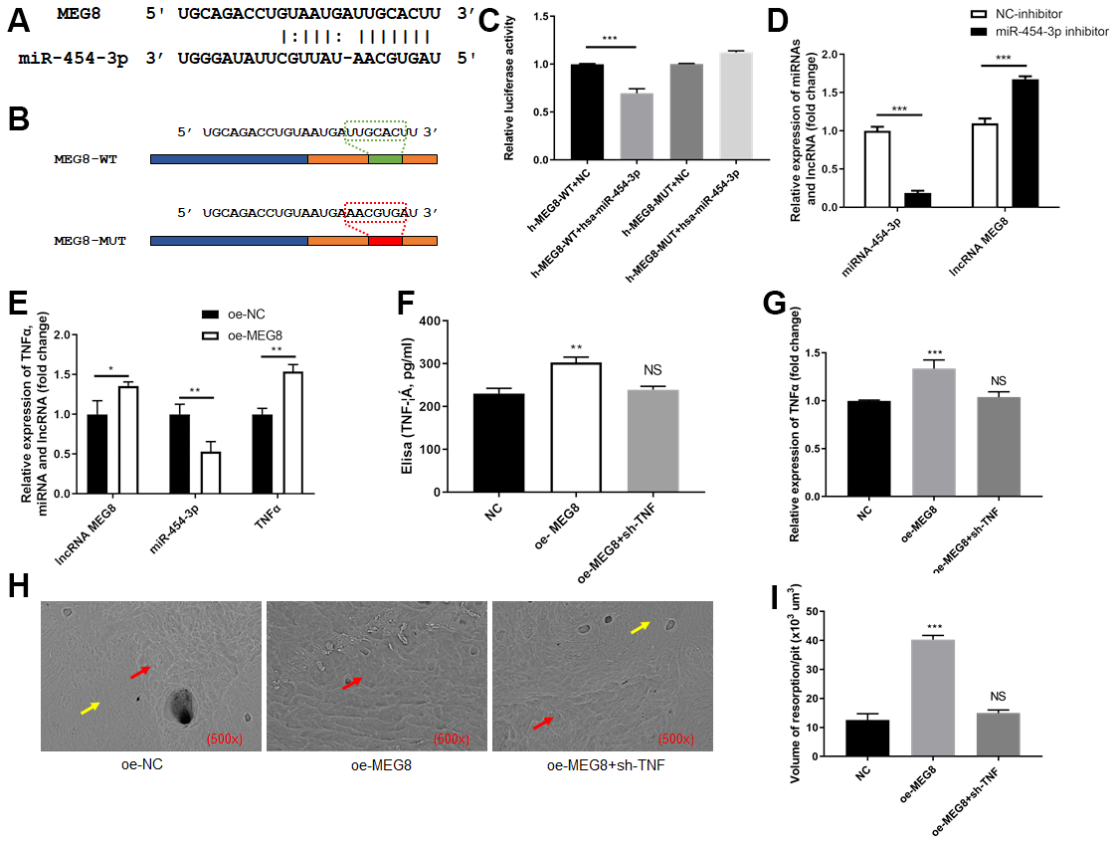
**MEG8 sponged miR-454-3p in 293T cells.** (**A**) The predicted binding site between MEG8 and miR-454-3p. (**B**) The predicted miR-454-3p binding site (MEG8-wt) and its matched mutant site (MEG8-mut). (**C**) Luciferase activity detection. (**D**, **E**) RT-qPCR analyses measured relative MEG8, miR-454-3p and TNF-α expression levels. (**F**) Elisa analyses measured TNF-α expression levels. (**G**) RT-qPCR analyses measured TNF-α expression levels. (**H**) Scanning electron microscope results of bone slices (500x). (**I**) Volume of resorption/pit (x10^3^ um^3^). *P < 0.05, **P < 0.01, ***P < 0.001.

Next, we attempted to explore the relationship between miR-454-3p and MEG8. It was predicted based on data from the StarBase and TargetScan. The predicted binding site between MEG8 and miR-454-3p was presented in Figure 4A. We constructed luciferase reporter plasmids containing the predicted miR-454-3p binding site (MEG8-wt) and its matched mutant site (MEG8-mut) (Figure 4B). Co-transfection of miR-454-3p and MEG8-wt dramatically reduced the luciferase activities, while co-transfection with miR-454-3p and MEG8-mut had no influence on the luciferase activities (p<0.001, Figure 4C).

### Inhibiting miR-454-3p increases the expression of MEG8

After miR-454-3p was knocked down by its inhibitor in 293T cells, the expression of MEG8 increased significantly (p<0.001, Figure 4D).

### Promoting MEG8 decreases the expression of miR-454-3p, increases the expression of TNF-α and further promotes RAW264.7 cells to destroy bone

After MEG8 was overexpressed in 293T cells, the expression of miR-454-3p decreased significantly (p<0.01, Figure 4E), the expression of TNF-α increased significantly (p<0.01, Figure 4E). ELISA also indicated that the amount of TNF-α in the medium increased after MEG8 was overexpressed (p<0.01, Figure 4F). We use cell and bone slices co-culture experiments to study the changes in cell's osteoclast ability. When MEG8 of 293T cells in the lower chamber of transwell was overexpressed, the expression of TNF-α increased, which resulted in the bone-breaking ability of RAW264.7 cells was enhanced in the upper chamber of transwell (Figure 4G), and the bone-breaking area of RAW264.7 cells was increased significantly (p<0.001, Figure 4H). Moreover, we added additional groups to conduct rescue experiments (Figure 4F, 4I). These results further confirmed that promoting MEG8 decreases the expression of miR-454-3p, increases the expression of TNF-α and further promotes RAW264.7 cells to destroy bone.

### Overexpressing MEG8 promotes progression of tumors through downregulating miR-454-3p and upregulating TNF-α *in vivo*

**Figure 5:**
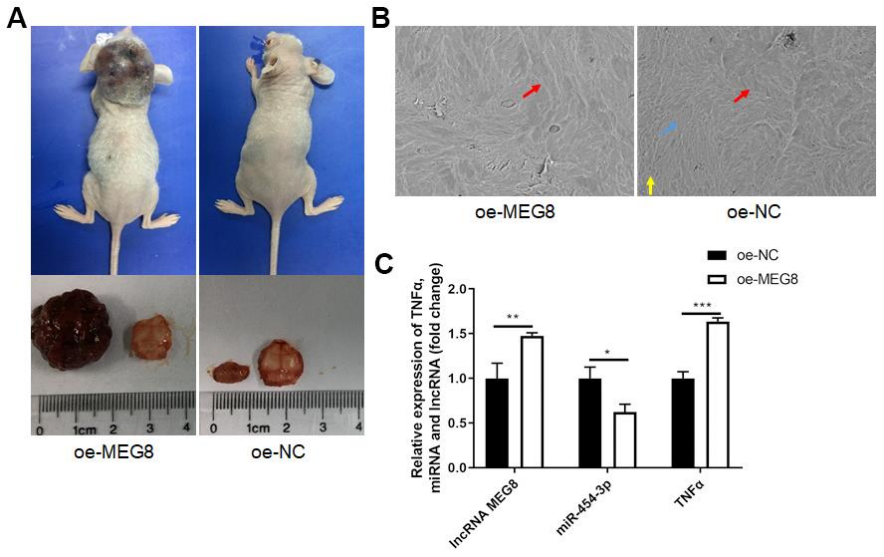
**Overexpression of MEG8 promoted tumorigenesis and tumor progression *in vivo*.** (**A**) Mice models, tumor volume and bone slices. (**B**) Scanning electron microscope results of bone slices (500x). (**C**) RT-qPCR analyses measured relative MEG8, miR-454-3p and TNF-α expression levels.

We established xenograft nude mouse models to elucidate the role of MEG8 *in vivo*. Results showed that MEG8 overexpressing significantly promoted xenograft tumor volume (Figure 5A) *in vivo*. Scanning electron microscope showed that MEG8 overexpressing promoted bone destruction (Figure 5B). MEG8 and TNF-α are upregulated in tumor tissues of oe-MEG8 group, while miR-454-3p is downregulated (Figure 5C). These results suggested that overexpressing MEG8 promotes progression of tumors through downregulating miR-454-3p and upregulating TNF-α *in vivo*.

### LncRNA MEG8 promotes TNF-α expression by sponging miR-454-3p in BIPAs

**Figure 6:**
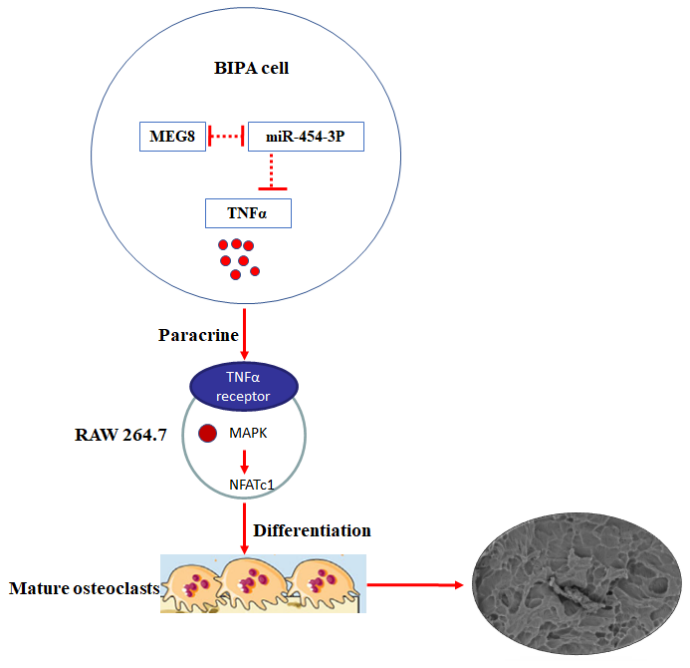
**Mechanism and function of TNF-α in BIPAs progress.** LncRNA MEG8 promotes TNF-α expression by sponging miR-454-3p, and then TNF-α directly induce osteoclast differentiation in BIPAs, which further leads to bone destruction.

Through *in vivo* and *in vitro* experiments, we have fully verified the MEG8/miR-454-3p/TNF-α ceRNA regulatory network (Figure 6). We found that lncRNA MEG8 promotes TNF-α expression by sponging miR-454-3p in BIPAs.

## DISCUSSION

In histology, pituitary adenomas are mostly benign tumors. But a considerable portion of pituitary adenoma is invasive. Pituitary adenomas may invade the cavernous sinuses, the bone and the sphenoid sinus. According to reports, invasive adenomas account for 45% of all pituitary adenomas [13, 14]. Invasive pituitary adenoma often results in low total resection rate and high recurrence rate, which seriously affects the quality of life of patients. There are many studies on invasive pituitary adenoma. At present, most research has focused on pituitary adenomas which invade cavernous sinus or diaphragma sellae. However, the clinical characters and the molecular mechanism of BIPAs are unclear because related research is rare. In our previous study, multivariate analysis showed that invasion was an independent risk factor for pituitary adenoma regrowth [1].

We firstly reported that the expression level of TNF-α in BIPAs were significantly higher than that in NIPA and TNF-α can directly induce osteoclast differentiation, which further leads to bone destruction [1]. TNF-α is a pleiotropic cytokine that is induced in response to a variety of pathological and physiological processes [15]. Studies found that TNF-α is closely related to the malignant behavior of the tumor. TNF-α may serve as a prognostic marker for chordoma progression because TNF-α increased the migration and invasion of chordoma cells [16]. TNF-α-induced MMP-9 secretion from mesothelial cells plays an important role in the metastatic dissemination of gastric cancer [17]. In addition, TNF-α has played a key role in the progression of breast cancer [18], colorectal cancer [19] and cervical cancer [20]. In the current study, we further confirmed that TNF-α was highly expressed in BIPAs and increased the risk of regrowth.

We speculated that lncRNA MEG8, miR-454-3p and TNF-α can constitute a ceRNA regulatory network through transcriptome microarray analysis and cell experiments in previous study [1]. More evidence demonstrates that lncRNAs play important roles in cancer initiation and progression by serving as ceRNAs through competitively binding to shared miRNAs [21]. There are more and more studies on ceRNA regulatory network of tumor [22–24]. In our study, we have fully verified that MEG8 promotes TNF-α expression by sponging miR-454-3p in BIPAs.

There are relatively few studies on lncRNA MEG8. It has been confirmed that MEG8 was over-expressed in activated hepatic stellate cells, injured hepatocytes and fibrotic livers [25]. In colorectal cancer, MEG8 may have a regulatory role in the adenoma-carcinoma transition [26]. MiRNAs are a class of noncoding RNAs that regulate gene expression by binding to their target mRNA. For instance, miR-454-3p promotes proliferation and induces apoptosis in human cervical cancer cells by targeting TRIM3 [27]. Similarly, miR-454-3p exerts tumor-suppressive functions by down-regulation of NFATc2 in glioblastoma [12]. Our data shows that MEG8 is upregulated in BIPA tissues while miR-454-3p is downregulated. We further proved that MEG8 and TNF-α can competitively combine with miR-454-3p.

Through *in vivo* and *in vitro* experiments, we have fully verified the MEG8/miR-454-3p/TNF-α ceRNA regulatory network in BIPAs. The functional assays revealed that MEG8/miR-454-3p targets TNF-α and forms the MEG8/miR-454-3p/TNF-α axis to regulate the initiation and progression of BIPAs.

## CONCLUSIONS

We firstly reported that TNF-α expressions were increased in BIPA tissues. Highly expressed TNF-α in BIPAs was associated with poor prognosis. Mechanically, we revealed a novel MEG8/miR-454-3p/TNF-α ceRNA regulatory network in BIPAs. Combined with our previous research, the mechanism of TNF-α affecting pituitary adenoma to invade bone is more perfect. LncRNA MEG8 promotes TNF-α expression by sponging miR-454-3p, and then TNF-α directly induce osteoclast differentiation in BIPAs, which further leads to bone destruction (Figure 6). It may provide a useful strategy for diagnosis and treatment for BIPA patients.

## MATERIALS AND METHODS

### Clinical materials and tissue specimens

A total of forty pituitary adenoma specimens (twenty BIPAs and twenty NIPAs) which were obtained in the operation from January 2008 to December 2015 in Beijing Tiantan Hospital, Capital Medical University. This study was approved by the Beijing Tiantan Hospital Ethics Committee, and every participant signed an informed consent form. Classification depends on preoperative images (Figure 1A), bone destruction observed during operation or bone tissue infiltration showed on the pathological reports. The pituitary adenoma specimens were obtained at the time of transsphenoidal surgery and quickly frozen in liquid nitrogen and fixed in a 10% formaldehyde solution within 30 minutes after the operation. The details of the patients are presented in Table 1.

**Table 1:** The details of the patients.

**NO.**	**Sex**	**Age**	**Operative time**	**Diameter(mm)**	**Resection**	**Volume(mm^3^)**	**Tumor type**
1	female	43	2015/5/20	≤30	GTR	12425.98	NIPA
2	female	25	2014/7/8	≤30	GTR	1061.424	NIPA
3	male	43	2012/7/16	≤30	GTR	3506.832	NIPA
4	female	35	2015/1/21	≤30	GTR	1573	NIPA
5	female	42	2015/8/4	≤30	GTR	2387.715	NIPA
6	male	30	2013/4/22	≤30	NGTR	7674.762	NIPA
7	female	49	2012/12/26	≤30	GTR	4382.736	NIPA
8	male	53	2013/1/28	≤30	GTR	2451.772	NIPA
9	male	37	2013/1/29	≤30	GTR	7771.104	NIPA
10	male	52	2013/3/12	≤30	GTR	7539.675	NIPA
11	male	45	2013/5/3	≤30	GTR	1597.824	NIPA
12	male	61	2013/5/6	≤30	NGTR	3202.668	NIPA
13	male	35	2013/6/3	≤30	GTR	11268.4	NIPA
14	male	31	2013/6/3	≤30	GTR	2424.98	NIPA
15	female	54	2013/7/4	≤30	GTR	3336.84	NIPA
16	male	42	2013/7/4	≤30	GTR	13319.56	NIPA
17	male	74	2013/7/30	≤30	GTR	7248.45	NIPA
18	male	47	2013/8/7	≤30	GTR	2277.882	NIPA
19	female	29	2013/8/27	≤30	NGTR	14134.29	NIPA
20	female	58	2013/9/18	>30	GTR	14040.97	NIPA
21	female	57	2014/7/30	>30	NGTR	50737.06	BIPA
22	female	67	2013/1/7	>31	GTR	55522	BIPA
23	female	41	2014/5/6	>30	GTR	19125.29	BIPA
24	male	43	2015/4/7	>30	NGTR	110705.6	BIPA
25	female	44	2015/10/10	>30	NGTR	21000	BIPA
26	female	46	2012/11/14	>31	NGTR	51226.56	BIPA
27	female	45	2012/12/6	>30	NGTR	100670.9	BIPA
28	female	61	2012/12/27	>30	NGTR	69888	BIPA
29	male	45	2013/1/30	>30	NGTR	41347.22	BIPA
30	male	14	2013/4/11	>30	NGTR	72540	BIPA
31	male	33	2013/4/17	>30	NGTR	60819.2	BIPA
32	female	60	2013/5/27	>30	NGTR	22510.03	BIPA
33	female	62	2013/12/2	>30	NGTR	120010.2	BIPA
34	male	43	2013/11/27	>30	NGTR	62128.05	BIPA
35	female	43	2013/11/21	>30	GTR	59518.8	BIPA
36	female	63	2014/1/20	>30	NGTR	87360	BIPA
37	female	47	2014/2/8	>30	NGTR	207090	BIPA
38	male	43	2014/2/26	>30	NGTR	49771.65	BIPA
39	male	59	2014/3/6	>30	GTR	56606.63	BIPA
40	male	49	2014/6/26	>30	NGTR	27363.74	BIPA

*GTR, Gross total resection; NGTR, Non-gross total resection; NIPA, non-invasive pituitary adenoma; BIPA, bone-invasive pituitary adenoma.

### Luciferase activity detection

To construct a luciferase reporter vector, TNF-α-3'UTR fragments and MEG8 cDNA fragments that containing the miR-454-3p binding site were inserted into the pGL3 plasmids. The TNF-α-3'UTR-MUT fragment and the MEG8-MUT fragment with the miR-454-3p mutated binding site were also inserted into the pGL3 vector. Using the Lipofectamine 3000 (Invitrogen, USA), recombinant plasmids (pGL3-MEG8, pGL3-MUG8-MUT, pGL3-TNF-α-3'UTR, pGL3-TNF-α-3'UTR-MUT) and Renilla reference plasmid were respectively co-transfected with miR-454-3p mimic or NC-mimic into HEK293T cells. After48 hours, luciferase activity was determined using a luciferase assay kit (BioVision Technologies, USA) and a luciferase reporter assay system (Promega, USA). With Renilla luciferase as the internal reference, the ratio of the relative luciferase unit value of firefly luciferase to that of Renilla luciferase shows whether the target reporter gene is activated.

### Cell lines and cell culture

293T and RAW264.7 cells were authenticated by China Infrastructure of Cell Line Resource. And they were tested negative for mycoplasma by China Infrastructure of Cell Line Resource. 293T cells were cultured in DMEM (American Type Culture Collection, USA) high glucose medium with 10% fetal bovine serum (FBS) (Gibco, USA), 2mM L-glutamine (Thermo Fisher Scientific, USA) and 1% penicillin/streptomycin (Thermo Fisher Scientific, USA). RAW264.7 cells were cultured in DMEM (American Type Culture Collection, USA) high glucose medium with 10% FBS (Gibco, USA) and 1% penicillin/streptomycin (Thermo Fisher Scientific, USA). All cells were cultured in a humidified incubator containing 5% CO2 at 37° C.

### Cell treatment and transfection

The 293T cells were transfected with the following sequences, plasmids and shRNA recombinant lentiviral: miR-454-3p inhibitor (RIBOBIO, China), MEG8 overexpression plasmid (RIBOBIO, China) (oe-MEG8), TNF shRNA recombinant lentiviral (RIBOBIO, China) (sh-TNF) as well as corresponding negative controls (NC-inhibitor and oe-NC). 293T cells were seeded in 6-well plates at a cell seeding density of 5×10^5^ cells/well. After cell density had reached 80%, transfection was performed in accordance with the instructions of the Lipofectamine 3000 kit (Invitrogen, USA). The cells and cell culture were harvested for the subsequent experiments after forty-eight hours.

### Cell and bone slices co-culture

To establish a co-culture system between 293T cells and bone slices (mouse skulls) with RAW264.7 cells, 5 × 10^5^ 293T cells were seeded at the bottom of six well plates, while 5 × 10^5^ RAW264.7 cells and one bone slice were added into the upper Transwell insert (0.4 μm pore size, Corning, USA). After the co-culture system was maintained with complete medium for seven days, the bone chips were taken out for the subsequent experiments.

### Xenograft experiments

A 293T cell xenograft model was performed for *in vivo* assays. This study was approved by the Beijing Tiantan Hospital Ethics Committee. 20 male BALB/c nude mice (6 weeks old) were randomly divided into two groups and given 3×10^6^ either oe-MEG8 transfected or oe-NC transfected 293T cells with serum-free medium by subcutaneous injection on top of their head. Four weeks after the cells were injected, the mice were euthanized, tumor and mouse skulls were taken out for subsequent experiments.

### RNA extraction and RT-qPCR

Total RNA was extracted from human pituitary adenoma tissues, 293T cells and xenograft tumor in nude mice using with TRIzol reagent (Invitrogen, USA). We used High-Capacity cDNA Reverse Transcription Kit (Thermo Fisher Scientific, USA) to perform reverse transcription according to the manufacturer’s instructions. The primer sequence is shown in Table 2. Quantitative PCR (qPCR) was performed and relative quantities of each gene were analyzed according to previous research.

**Table 2:** The primers used for RT-qPCR with their sequence.

**Gene**	**Forward primer (5’–3’)**	**Reverse primer (5’–3’)**
MEG8	TGCACTTTGCTGATTGAAGG	TCTCCAGGCTCCATCCTAAA
miR-454-3p	ACCCTATCAATATTGTCTCTGC	GCGAGCACAGAATTAATACGAC
TNFα	AAACAATGCTGATTTGGTGAC	GCAAACTTTATTTCTCGCCACT
GAPDH	TGACTTCAACAGCGACACA	CACCCTGTTGCTGTAGCCAAA

### Protein extraction and western blot

Tumor tissues were lysed with RIPA buffer (Applygen, China) containing a mixture of protease inhibitors (Applygen, China). We used the BCA Protein Assay kit (Thermo Fisher Scientific, USA) to determine the protein concentration. Western blot experiments were carried out with reference to previous studies. The primary antibodies and secondary antibodies used in the experiment are shown in Table 3.

**Table 3:** The antibodies used in the experiments.

**Antibody**	**No.**	**Company**	**Country**
Anti-TNFα antibody	ab6671	Abcam	Britain
Anti-GAPDH antibody	K200057M	Solarbio	China
Anti-Rabbit antibody	ZB-2301	ZSGB-BIO	China

### HE staining and IHC

The pituitary adenoma specimens were fixed in 10% paraformaldehyde, decalcified in formic acid, and embedded in paraffin. All samples were sectioned at 5 mmol/L. Pituitary adenoma specimens were stained with HE and processed for IHC staining with anti-TNF-α (ab 8348, 1:1,200, Abcam).

### Enzyme-linked immunosorbent assay (ELISA)

The levels of TNF-α in cell culture supernatant were determined using a human TNF-α Quantikine ELISA Kit (Minneapolis, USA) according to manufacturer’s instructions.

### Scanning electron microscope

The bone slices were placed in NH_4_OH for half an hour. Then they were sonicated for 10 minutes to remove surface cells. They were subjected to gradient alcohol dehydration, naturally air-dried, and coated with gold powder. Finally, they were examined by scanning electron microscope.

### Statistical analysis

SPSS 25.0 statistical software was used for statistical evaluation. According to the grouping situation, statistical analysis of the data was performed using one-way ANOVA or independent two-sample t tests. The regrowth-free curves were generated by the Kaplan–Meier method. Correlation was evaluated using Pearson’s correlative analysis. P<0.05 were defined as a significant difference.

### Limitation

In this study, we tried to use primary cells for functional experiments. But we did not achieve good results. The reason is that pituitary adenomas are benign tumors. Under our existing primary culture technology, they cannot maintain a good growth state, and thus cannot meet the functional experimental requirements of this study. Therefore, in this study, we have to use 293T cells for functional experiments. Our team is working hard to solve the problem of primary culture of pituitary adenoma.
